# Diet and Human Mobility from the Lapita to the Early Historic Period on Uripiv Island, Northeast Malakula, Vanuatu

**DOI:** 10.1371/journal.pone.0104071

**Published:** 2014-08-20

**Authors:** Rebecca Kinaston, Stuart Bedford, Michael Richards, Stuart Hawkins, Andrew Gray, Klervia Jaouen, Frederique Valentin, Hallie Buckley

**Affiliations:** 1 Department of Anatomy, Otago School of Medical Sciences, University of Otago, Dunedin, New Zealand; 2 Department of Archaeology and Natural History, College of Asia and the Pacific, Australian National University, Canberra, ACT, Australia; 3 Department of Anthropology, University of British Columbia, Vancouver, Canada; 4 Department of Human Evolution, Max Planck Institute for Evolutionary Anthropology, Leipzig, Germany; 5 School of Archaeology and Anthropology, The Australian National University, Canberra, ACT, Australia; 6 Department of Preventive and Social Medicine, Otago School of Medical Sciences, University of Otago, Dunedin, New Zealand; 7 CNRS, Maison de l’Archéologie et de l’Ethnologie, Nanterre, France; Ohio State University, United States of America

## Abstract

Vanuatu was first settled ca. 3000 years ago by populations associated with the Lapita culture. Models of diet, subsistence practices, and human interaction for the Lapita and subsequent occupation periods have been developed mainly using the available archaeological and paleoenvironmental data. We test these models using stable (carbon, nitrogen, and sulfur) and radiogenic (strontium) isotopes to assess the diet and childhood residency of past communities that lived on the small (<1 km^2^) island of Uripiv, located off the northeast coast of Malakula, Vanuatu. The burials are from the initial Lapita occupation of the island (ca. 2800–2600 BP), the subsequent later Lapita (LL, ca. 2600–2500 BP) and post-Lapita (PL, ca. 2500–2000 BP) occupations, in addition to a late prehistoric/historic (LPH, ca. 300–150 BP) occupation period. The human stable isotope results indicate a progressively more terrestrial diet over time, which supports the archaeological model of an intensification of horticultural and arboricultural systems as local resources were depleted, populations grew, and cultural situations changed. Pig diets were similar and included marine foods during the Lapita and PL periods but were highly terrestrial during the LPH period. This dietary pattern indicates that there was little variation in animal husbandry methods during the first 800 years of prehistory; however, there was a subsequent change as animal diets became more controlled in the LPH period. After comparison with the local bioavailable ^87^Sr/^86^Sr baseline, all of the Lapita and LPH individuals appeared to be ‘local’, but three of the PL individuals were identified as “non-local.” We suggest that these “non-locals” moved to the island after infancy or childhood from one of the larger islands, supporting the model of a high level of regional interaction during the post-Lapita period.

## Introduction

**Figure 1:**
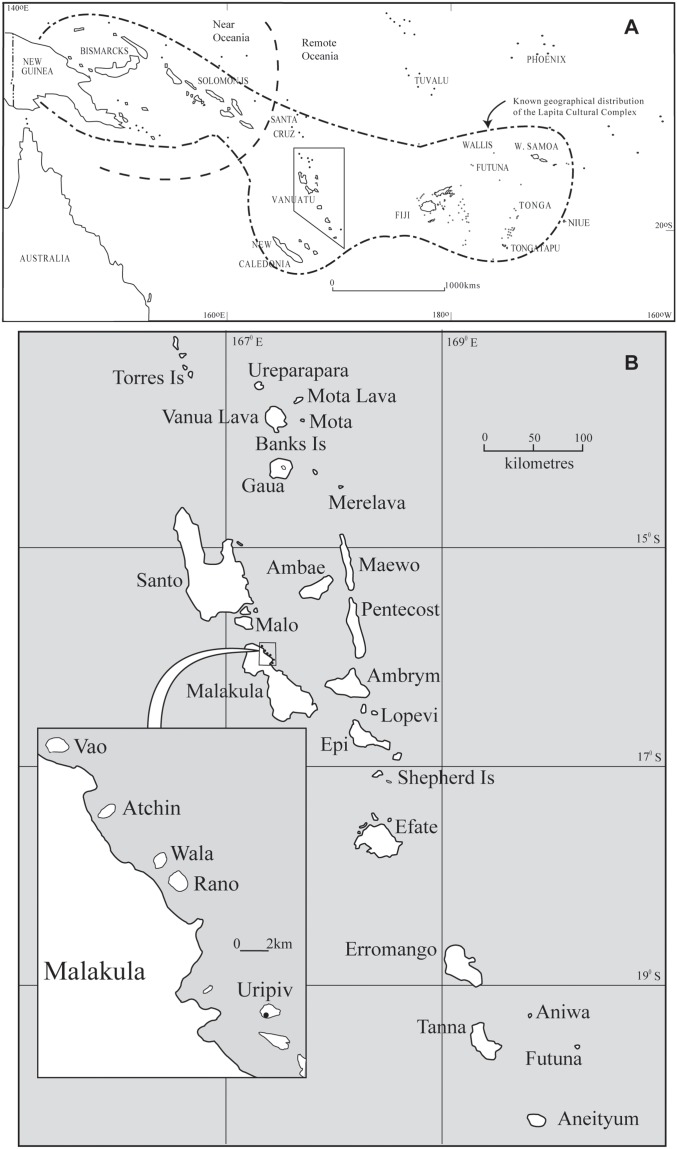
Map of the Pacific Islands (A) and the location of Uripiv in Vanuatu (B). In Figure 1A, the short dashed line designates the boundary between Near and Remote Oceania and the continuous dashed line represents the known distribution of the Lapita Cultural Complex.

Around 3300 BP an Austronesian-speaking people migrated eastward from Island South East Asia (ISEA) and established settlements on the previously inhabited islands of Papua New Guinea and the Solomon Islands, which can be identified today from remnants of the Lapita culture [1]–[4]. Lapita populations rapidly continued their easterly movement, entering the uninhabited islands of Remote Oceania around 3100 BP (Fig. 1). This colonization of Remote Oceania marked one of the last great human migrations on earth [5], [6].

Analyzing the level of interaction between and within these colonizing groups has been a major focus of archaeologists working in the region. Source analyses of stone tools-especially those made of obsidian - and pottery vessels have established that, in some cases, these items were transported over long-distances during early Lapita times [5], [7]–[10]. Soon after initial settlement across the Lapita distribution (from New Guinea to Samoa) there is evidence of increasing regionalization and diversification in material culture and languages [8], [9], [11], [12]. There is still some evidence of diluted connections across the Lapita distribution in the form of decorative motifs on pottery from the late Lapita period, but diversification seems to dominate the material culture. Localized networks became increasingly important in the post-Lapita period as populations grew and settlements became firmly established and adapted to diverse landscapes [7], [13].

The analysis of radiogenic isotopes within tooth tissues has the potential to provide information about mobility within prehistoric Pacific island populations [14]–[17]. Strontium isotopes can be used to identify individual migrants within a burial population, which can provide clues about population interactions and social organization, such as matrilocality [18]–[20]. Strontium isotope analyses of humans and pigs from Watom Island in the Bismarck Archipelago of New Guinea (ca. 2700–2500 BP) [15], [16] and the earliest human settlers of Vanuatu, interred in the burial ground of Teouma, Efate Island, Vanuatu (ca. 3000 BP) [14], have confirmed that this method is a useful way to identify non-local animals and people from Lapita contexts in both Near and Remote Oceania.

The subsistence regime and diet of Lapita communities would have been important for the success of initial and subsequent settlements. Lapita populations likely brought with them a ‘transported landscape’ of domestic animals and cultivars that were essential to their success as they adapted to exotic island environments, causing the mass depletion and extinction of plants and animals as they moved eastward across the Pacific Ocean [2], [21], [22]. Generally, current archaeological thought supports the theory that Lapita subsistence involved broad-spectrum foraging of marine and terrestrial resources coupled with horticulture and animal husbandry [23]–[29]. The stable isotope analysis of human and animal bone collagen has the potential to directly assess prehistoric diet, potentially clarifying interpretations regarding food consumption patterns and subsistence strategies during the Lapita settlement of Remote Oceania and later post-Lapita periods. For example, stable isotope analysis can shed light on the relative importance of domestic and wild protein resources at each site, intra-population dietary variation, and temporal variation in diet between the Lapita and post-Lapita periods, and can potentially provide evidence of animal husbandry practices [30], [31].

Stable isotope analysis has been used to reconstruct the palaeodiet of humans interred in a late Lapita site on Watom Island, East New Britain, New Guinea [24], [32]–[35], an early Lapita burial ground at Teouma, Efate Island, Vanuatu [28], [29], [36], a wide range of prehistoric periods from sites on the Lau Islands, Fiji (beginning with the Lapita period) [37] and post-Lapita and later sites on Waya Island and Sigatoka, Fiji [34], [38]. With the exception of Teouma, these analyses were conducted on less than six individuals (e.g. Watom) or skeletal elements not associated with interments (e.g. Fijian sites). Due to the small size and limited temporal spread of these assemblages, these studies have not been able to assess potential dietary transitions from the Lapita to post-Lapita (and later) periods at any one site using intact burials from secure contexts.

The archaeological site on Uripiv Island, northeast Malakula, Vanuatu, contains burials that date to the later Lapita (ca. 2800–2500 BP), the post-Lapita (ca. 2500–2000 BP), and late prehistoric/historic (ca. 300–150 BP) occupation periods in Vanuatu [39]. The initial Lapita occupation of Uripiv began approximately 200 years after the first Lapita colonists (i.e. early Lapita) had arrived in Vanuatu [6], [40], and therefore all evidence of Lapita occupation on Uripiv can be considered technically middle/late Lapita in the prehistoric sequence of the archipelago. The burials recovered from Uripiv date to the initial Lapita occupation phase of the island (layer 4, n = 7, ca. 2800–2600 BP), the later Lapita (LL) occupation phase (layer 3, n = 3, ca. 2600–2500 BP), the post-Lapita (PL) occupation phase (layer 2, n = 23, ca. 2500–2000 BP) and the late prehistoric/historic (LPH) occupation phase (layer 1, n = 5, ca. 300–150 BP). As two distinct Lapita layers have been identified on Uripiv, we use the terms ‘Lapita’ to describe the burials from the initial occupation of the island (layer 4) and ‘later Lapita’ (LL) to describe the burials from layer 3. While small by global standards, the Uripiv skeletal sample (n = 38) constitutes the largest assemblage of burials spanning these periods yet discovered at a single site in the Pacific Islands and includes a number of infants and young children. In the context of the comprehensive dietary and strontium isotope baselines established in this study, the isotopic analyses of humans and pigs from Uripiv provide a unique opportunity to assess diet, subsistence strategies, methods of animal husbandry, and human mobility during these temporal periods.

This study has two aims. The first is to use stable isotope analysis to assess if there was a transition to more plant foods from the Lapita to the post-Lapita and late prehistoric/historic periods for both humans and pigs. As the archaeological models predict, this transition would indicate a heavier reliance on horticultural products as human and domestic animal populations grew larger, local wild resources were depleted, and cultural diversification occurred. Our second aim is to use strontium isotope ratios to assess patterns of human mobility at the site, as this has proven difficult to determine using other lines of evidence (e.g. material culture and linguistic analyses).

### The site and skeletal sample

#### The site

In 2001 a Lapita settlement site was found on the small island of Uripiv, off the northeast coast of Malakula in Vanuatu (Fig. 1). The pottery at the site was indicative of Lapita with the distinctive dentate stamped designs and vessel shapes, and some incised pottery [41], [42]. Evidence for continued post-Lapita occupation (ca. 2500–2000 BP) was identified extending across a much larger area of the site. Lapita and post-Lapita burials were discovered during subsequent excavations at the site in 2001 and 2002, and a return to the site in 2005 with bioarchaeologists confirmed an extensive burial area. Various excavations around the island revealed a change in settlement patterns after the eruption of the Ambrym volcano around 1800 BP. More extensive excavations centered around the burial ground on Uripiv were conducted during 2009–2011, yielding a further 30 burials from various contexts (i.e. the Lapita, LL, PL, and LPH layers). Only eight of the burials from Uripiv have been fully detailed in a previous publication [39] and no isotopic analyses from the site have been published before now.

Human and animal bones from Uripiv, especially within the earliest Lapita layers, were morphologically well-preserved as a result of subsequent midden dumping and transported coral gravel surfaces, volcanic tephra, and sand from cyclonic and storm events [39]. Five archaeological layers were identified at Uripiv: the sterile sand beach (layer 5); the initial Lapita-associated occupation and burials located directly on top of and cut into the sterile beach (layer 4); a dark brown sandy layer associated with the later Lapita midden and burials (layer 3); a branch coral and pebble floor layer from the post-Lapita occupation (including burials) of the site (layer 2); and finally, a dark brown volcanic tephra layer (layer 1) where late prehistoric/historic burials were found at its lower levels [39]. The volcanic ash component of the site is primarily derived from nearby Ambrym, where major eruptions date from 1800 BP [43], [44].

#### The ecology of Uripiv and the archaeological evidence of prehistoric diet at the site

Before human settlement the vegetation of northern Malakula, Vanuatu, would have consisted of lowland rainforest and coastal plants, including mangroves. During the Lapita settlement of Vanuatu, the climate may have been drier than it currently is in modern times as a result of an increase in El Niño Southern Oscillation (ENSO) events [45]. Today, most of mainland Malakula and the offshore islands, including Uripiv, have been extensively modified by humans and are characterized by gardens or secondary forest [46]. As there is no running water on Uripiv, water must be obtained from rainwater collection or by digging a well to utilize the freshwater in the Ghyben-Herzberg lens [41]. The Ghyben-Herzberg lens is tapped for limited agricultural purposes, but the modern day inhabitants of Uripiv cultivate their primary gardens on the adjacent Malakula mainland [47] and oral traditions suggest that this has been a pattern for many generations.

The identification of microfossils from exotic cultivated plant species within soil samples and pottery residues indicate that horticulture was practiced from the time of the initial Lapita settlement of Uripiv [44], [47]. Analyses of starch grains, calcium oxalate crystals, and xylem cells identified plants from the Araceae family (taro), which were attributed to some type of introduced non-*Colocasia* aroid (*Alocasia macrorrhiza, Amorphophallus paeoniifolius,* and *Cyrtosperma merkusii*) [47]. Although *Amorphophallus* spp. grow wild in Vanuatu today, the plant is thought to have been brought by humans sometime in the past (V. Lebot pers. comm.). Further phytolith analysis has revealed that banana (*Musa* sp.) was also present on Uripiv during the Lapita period [44]. The presence of these taxa in Lapita layers on Uripiv “supports the indirect evidence (e.g. permanent settlements) that horticulture was a significant part of the Lapita Cultural Complex” ([44]:2053). A recent analysis of microfossils in the dental calculus of both Lapita and post-Lapita individuals from Uripiv has identified *Musa* sp. phytoliths and material that may be starch granules from exotic root vegetables such as aroids, a species of yam (*Dioscorea esculenta*), and arrowroot (*Tacca leontopetaloides*), providing further evidence of the presence of horticulture on Uripiv since the initial Lapita colonization of the island [48]. No direct evidence for prehistoric arboriculture has been identified on Uripiv (e.g. the remains of *Barringtonia edulis* and *Canarium* spp.), but cultivated nuts and fruits provide important food resources throughout the Western Pacific today and were likely part of the transplanted landscape of the Lapita populations [2], [49]–[51].

Based on an initial analysis of the Uripiv faunal material, it is clear that both wild and domestic/commensal animal species were eaten from the time of initial Lapita settlement [41]. Today, the native terrestrial fauna of Vanuatu is comprised primarily of birds and bats but also includes a number of species of small Squamate lizards [52]. A pattern of extinction, extirpation, and reduction in wild taxa has been observed at a number of other Lapita sites throughout the Pacific Islands [22], [53] and likely also caused a rapid decline in these wild taxa on small islands such as Uripiv. It is important to note that, due to a biogeographic disjunction in the island group, mainland Malakula has a larger diversity of terrestrial fauna compared with the southern islands of Vanuatu [54] and may have provided the inhabitants of Uripiv further opportunities to hunt wild fauna.

Uripiv is surrounded by a fringing reef and there is a seagrass meadow on the western side of the island. Adjacent to Uripiv is the island of Uri, which is predominantly covered by a large mangrove forest surrounded by a fringing reef and, like Uripiv, also harbors a seagrass meadow. Seagrass meadows are feeding grounds for dugongs (*Dugong dugon*), marine turtles (*Chelonia mydas* and *Eretmochelys imbricata*), echinoderms, and a variety of shellfish species [55]. Mangroves provide a specialized niche for a number of taxa including edible shellfish, crustaceans, bats, and birds [56]. Coral reefs sustain a diverse array of fish, shellfish, crustaceans, and edible invertebrates such as echinoderms and octopi [57]. Ongoing and not yet completed analyses (S. Hawkins, R. Ono, S. Bedford and Y. Philip unpublished data) are revealing that species from all of these ecosystems are represented in the faunal remains that have been assessed from the site. With regard to shellfish, the dramatic decrease in species diversity and size indicates the heavy exploitation of these resources [42]. Scombrid fish remains and items of fishing technology found at the site provide strong evidence that pelagic fishing was practiced from canoes, probably in the deep ocean trench between Uripiv and the Malakula mainland.

### Reconstruction of diet and mobility patterns on Uripiv

#### Stable isotope analysis for paleodietary reconstruction

The analysis of the stable isotope ratios of carbon, nitrogen, and sulfur within human and animal bone collagen has become a routine procedure for the assessment of prehistoric diet [58]–[60]. Stable isotopes are measured in ratios (^13^C/^12^C, ^15^N/^14^N, and ^34^S/^32^S) in relation to an international standard (VPDB for carbon, AIR for nitrogen, and VSNOW for sulfur), which is expressed in per mil (‰) and designated by the delta symbol (δ). The stable isotope ratios of bone collagen in cortical bone are representative of at least the last ten years of (mostly) the protein diet of adults, but represent less time for infants and children due to the elevated rate of bone modeling and remodeling in growing individuals [61], [62].

Carbon stable isotope ratios (δ^13^C) are used to determine the consumption of plants with differing photosynthetic pathways (C_3_, C_4_, and CAM) and to differentiate between the consumption of organisms from marine and terrestrial environments [60]. Marine systems and C_4_ plants typically display higher δ^13^C values compared with terrestrial systems and C_3_ plants respectively, and although there are few edible CAM plants, they typically display δ^13^C values between those of C_3_ and C_4_ plants [63].

Nitrogen stable isotope ratios (δ^15^N) reflect the trophic position of an organism. With every trophic step there is an increase in δ^15^N values of 2–4‰, although this may be larger in humans (∼6‰) [64], [65]. As a result of observable trophic differences between organisms, δ^15^N values can help differentiate between the consumption of plants (lower values) and animals from higher trophic levels than plants (higher values) [60]. Freshwater and marine systems display higher δ^15^N values compared with terrestrial systems as they have longer food chains and consequently more trophic enrichment. Therefore, used in conjunction with δ^13^C values, δ^15^N values can help discern between the proportion of aquatic (marine and freshwater) and terrestrial foods in the diet [66], [67]. N_2_-fixing plants such as legumes and organisms from certain environments with a prevalence of N_2_-fixing bacteria, such as coral reefs and mangroves, display low δ^15^N values and must be taken into consideration when assessing potential dietary sources [68]–[70].

During breastfeeding, the nursing child is one trophic level higher than their mother, and its bone collagen is enriched in the heavy stable isotope of nitrogen (^15^N) by about 2–4‰ [71], [72]. A number of studies have assessed patterns of breastfeeding and weaning in prehistoric societies using the δ^15^N values of infant/young child bone collagen and tooth dentine, which forms during childhood [71], [73]. It is assumed that fetal (8 weeks in utero to birth) and neonatal (birth to 27 days old) individuals would not survive long enough to display a ‘breastfeeding signal’ and should display similar δ^13^C and δ^15^N values to those of their mothers, although exceptions to this have been observed [36].

Terrestrial sulphur stable isotope ratios are a reflection of the underlying geology, but because terrestrial δ^34^S values are consistently lower than marine systems (seawater displays a constant δ^34^S value of ∼20.0‰), they can also be used in conjunction with carbon and nitrogen stable isotope ratios to assess marine vs. terrestrial food consumption patterns [74]–[76]. It must be noted that the δ^34^S values of coastal terrestrial food webs may become elevated as a result of sea spray and marine-derived precipitation, a phenomenon known as the ‘sea spray effect’ [59], [77]. Pesticides, herbicides, fertilizers, and sulfur pollutants may also affect modern environmental δ^34^S values [59], [78]. However, the areas of Vanuatu where the modern samples were collected for this study are not industrialized and the inhabitants of northeast Malakula (including Uripiv) practice traditional methods of horticulture (i.e. no use of pesticides etc.) which supports that the δ^34^S values of the modern samples are most likely a true reflection of the local soils.

The δ^13^C, δ^15^N, and δ^34^S values of human and faunal bone collagen from a single site are comparable, taking into account trophic level differences. The trophic level differences for nitrogen stable isotopes are mentioned above, but these differences are smaller for carbon and sulfur stable isotope ratios, about +0–2‰ for δ^13^C and <1.0‰ for δ^34^S values [59], [79]. Nitrogen and sulfur are present in amino acids and the stable isotope ratios of these elements are therefore only representative of the protein portion of the diet [80]. Carbohydrates, lipids, and protein all contain carbon. Although the carbon in dietary protein is preferentially routed to synthesize bone collagen, carbon from these other macronutrients may also be used [81]–[84]. If the diet is monoisotopic (e.g. all components are from a C_3_ terrestrial ecosystem), a diet-tissue spacing of +5 ‰ can be used for δ^13^C values, although the composition of the dietary macronutrients in the diet may result in variations in diet-tissue spacing values [81], [82], [85].

#### Strontium isotope analysis for the assessment of prehistoric human mobility

Strontium isotope analysis has been used to assess the mobility and migration patterns of prehistoric humans and animals from archaeological sites around the world [76], [86], [87], including the Pacific Islands [14]–[16], [88], [89]. This method is based on the premise that rocks display variable ^87^Sr/^86^Sr ratios depending on their type, age, and original rubidium (Rb) content. ^87^Sr is formed by the decay of ^87^Rb, which has a half-life of 4.88×10^10^ [18]. As a result, rocks with older formation dates will display more radiogenic ^87^Sr/^86^Sr ratios compared with their more modern counterparts [86].

Mineral weathering and erosion of the underlying bedrock, airborne dust (loess), atmospheric deposition, groundwater, and stream water are major contributors to the strontium in soil [18], [20]. As a result of these processes, the labile, or biologically available, ^87^Sr/^86^Sr ratios of the soil may vary from the ^87^Sr/^86^Sr ratio(s) of the underlying bedrock [18], [20]. For example, underlying bedrock composed of different rock types will contribute variable amounts of strontium (with different ^87^Sr/^86^Sr ratios) to the soil depending on the type of rock and the rate of weathering [20]. Sea spray and marine-derived precipitation can potentially alter the ^87^Sr/^86^Sr ratios of coastal soils nearer to that of seawater (i.e. 0.7092 during modern times and the prehistoric period covered in this study) [90]. The depth of the water source and recharge time may affect groundwater ^87^Sr/^86^Sr ratios [20]. Stream water ^87^Sr/^86^Sr ratios may also vary as a result of the origin of eroded sediments carried by rivers and elevation [14]. It is thus necessary to establish the local bioavailable ^87^Sr/^86^Sr signature of an area by analyzing plants or animals with restricted home ranges [20], [90]. Plants mark the entry point of strontium into the local ecosystem because they utilize strontium from the water and the leachable component of the soil without measurable fractionation [18]. Humans and animals incorporate strontium primarily from plants, as meat displays very low strontium concentrations as a result of biopurification (the discrimination against strontium in preference of calcium) at each successive trophic level [19].

Human tooth enamel is resistant to diagenetic alteration in the burial environment; therefore strontium purified from enamel should be representative of the biogenic strontium available during the time of tooth mineralization [91]–[93]. Strontium is substituted for calcium in the hydroxyapatite of bone and tooth enamel [20]. The ^87^Sr/^86^Sr ratio of tooth enamel reflects the biologically available strontium isotope signature of the food and drink a person consumed during the time of tooth mineralization [86]. The deciduous dentition begins mineralizing during the second trimester in utero, starting with the central incisor, followed by the first molar, lateral incisor, canine, and second molar. The crowns of most of the deciduous dentition are complete at about one year of age, but canines and second molars can take up to sixteen months to form [94]. With the exception of the third molar crown, which forms between the ages of 9.9 and 12.6 years, the crowns of the other permanent dentition form between the first and seventh years of life [94], although the age of tooth formation may vary between populations with different genetic backgrounds [95].

The ^87^Sr/^86^Sr ratios of the permanent dentition are representative of the place of childhood or early adolescent residence. The deciduous dentition contains at least some of the dietary strontium consumed by the mother during pregnancy, but may also reflect strontium obtained from the maternal skeletal reservoir, which may vary from the local strontium isotope signature if she immigrated to the area before becoming pregnant [96]. The proportion of strontium utilized by the fetus from each source (i.e. maternal diet vs. bone turnover during pregnancy) has only been estimated for humans [97], but animal models indicate that a large proportion of fetal strontium comes from the maternal diet [98], [99]. The proportion of strontium in breast milk that originates from the maternal diet or maternal skeleton is, to the authors’ knowledge, also unknown for humans, although a study using ^90^Sr as a tracer in dairy cows showed that most strontium within the analyzed cows’ milk was derived from the maternal cows’ diet [100].

#### Geological setting of Uripiv

Provenance studies using strontium isotope ratios are only possible in regions that display heterogeneous geological substrates [18], [86]. In the Western Pacific, the Andesite Line delineates the boundary between continental islands with older geological origins (more felsic rocks) and the relatively recently formed islands of the Central Pacific Basin (primarily mafic volcanic rocks) [101]. The Vanuatu Archipelago lies west of the Andesite Line and is composed of basaltic rock types, such as gabbros, tholeiitic lavas, and calk-alkaline basalts, which formed at various times (35 -<1 Ma) and display a relatively narrow range of ^87^Sr/^86^Sr ratios, between ∼0.7030–0.7045 [14], [102], [103]. The Vanuatu island arc was formed during the subduction of the Indo-Australian Plate and the Pacific Plate, caused by a combination of magmatism and ocean floor uplift [104]. As a result of differences in the subduction history of the Vanuatu island arc, there is a geological transition between the southern and central islands that corresponds with observable differences in their geological ^87^Sr/^86^Sr ratios: the volcanoes of the southern islands of Erromango, Tanna, Aneityum, and Futuna display ^87^Sr/^86^Sr ratios between 0.7030–0.7032, whereas volcanoes to the north (from Efate to Gaua, including Malakula) display more radiogenic ratios, between 0.7036–0.7043 [104]. The active uplifting of many of the islands of Vanuatu, including Northeast Malakula, has raised coral reef limestone around coastal areas and formed small islands such as Uripiv and nearby Vao, Rano, Atchin, and Wala [103]. On the northeast coast of Malakula this uplift has occurred at a rate of about 1 m every thousand years [105]. Since the uplift occurred relatively recently, this coral reef limestone should display ^87^Sr/^86^Sr ratios comparable to modern seawater (∼0.7092) [14]. As a result of differences in the underlying geology, the strontium isotope signature of the humans eating food grown on the limestone island of Uripiv should differ from humans subsisting in areas with underlying volcanic bedrock or mixture of volcanic and limestone bedrocks.

## Materials

### Ethics statement

All necessary permits were obtained for the described study, which complied with all relevant regulations. Research agreements were signed between S. Bedford, the Vanuatu Kaljoral Senta, and the Vanuatu Cultural Council, which included permission to excavate the site on Uripiv, Vanuatu. The permits for exporting the human and faunal remains from the Uripiv archaeological site were issued by the Vanuatu Kaljoral Senta and the Vanuatu Cultural Council (export permit numbers 11/VLI/04, 04/VLI/05, 08/VLI/09, and 09/VLI/2010). The prehistoric human remains are currently curated at the Department of Anatomy, University of Otago, Dunedin, New Zealand, until repatriation to Uripiv in 2015. The prehistoric faunal assemblage from Uripiv is curated by the School of Archaeology and Anthropology at the Australian National University, Canberra, Australia, with the exception of the excess bone left over from stable isotope analysis (n = 10), which is stored in the Department of Anatomy, University of Otago, Dunedin, New Zealand. Modern plant and animal samples from Vanuatu were collected on private land with permission from private landowners and chiefs. Only modern wild animals (no domestic species) were sampled and no protected species were collected. All samples imported from Vanuatu (modern samples) or Australia (prehistoric samples) to the transitional facility at the University of Otago, Dunedin, New Zealand, were sent under the MAF (Biosecurity New Zealand Ministry of Agriculture and Forestry) Permit to Import Restricted Biological Products of Animal Origin (2011 permit number 2011041970) and the Permit to Import Laboratory Specimens (2011 permit number 2011041930). The modern plant and animal material is currently stored in a PC2 laboratory at the Department of Anatomy, University of Otago, Dunedin, New Zealand. All previously unreported specimen numbers are detailed in full in Tables S1, S2, and S3 and all previously published specimens numbers are reported in Kinaston et al. [28].

### Prehistoric humans

Cortical bone from 30 individuals was sampled for the palaeodietary analysis, including those from the Lapita (n = 6), LL (n = 3), PL (n = 17) and LPH (n = 4) periods. The age and sex of the Uripiv individuals were assessed using standards set by Buikstra and Ubelaker [106] and Scheuer and Black [94]. The Uripiv skeletal sample included a number of infant and child burials that were unevenly spread across temporal periods. Infants and children comprised the majority of the Lapita interments (n = 7/9), whereas only 4/17 of the PL individuals and 1/4 of the LPH individuals were subadults.

One tooth from each of the fifteen individuals was sampled for strontium isotope analysis. The second and third permanent molars (n = 7) were preferentially sampled, however, first molars (n = 2) and a premolar (n = 1) were collected when the former were not available. First (n = 2) and second (n = 2) deciduous molars, and one deciduous lateral incisor were collected from the subadult individuals (Table S1).

### The dietary and strontium baselines: sampling strategy

In order to interpret the human stable isotope data, a dietary baseline was created using the carbon, nitrogen, and sulphur stable isotope ratios of prehistoric fauna and modern plants and animals from Vanuatu [28]. The prehistoric fauna analyzed included eleven prehistoric pig (*Sus scrofa*) bones sampled from the Uripiv site (Table S2) and previously published stable isotope results from twenty-two prehistoric Pteropodidae bones (megabats) from the site of Teouma on Efate Island, Vanuatu [28], [29].

During August and September 2011, modern plants (n = 68) and animals (n = 30) were collected from Efate Island and northeast Malakula, including Uripiv Island. The stable isotope values (δ^13^C, δ^15^N, and δ^34^S) from these modern species were used to create a dietary baseline for the interpretation of the human and pig diets on Uripiv and the Teouma site on Efate Island, Vanuatu [28]. The δ^13^C values of the modern plants and animals were corrected by +1.5‰ for terrestrial and +0.86‰ for marine systems to account for the global decrease in ^13^C after the Industrial Revolution (i.e. the Suess effect) [32].

Thirty plant samples were also collected from unfertilized gardens on Efate Island (n = 15), Uripiv (n = 6), and the adjacent Malakula Mainland (n = 9), in order to establish a geological strontium isotope baseline for the interpretation of the human strontium isotope ratios. To ensure that a wide range of labile ^87^Sr/^86^Sr ratios were represented, three samples-one from a short-rooted plant, one from a medium-rooted plant, and one from a deep-rooted plant-were collected from each sampling locality (Table S3).

## Methods

Approximately 1.5 g of cortical bone from the long bones of the adult humans and pigs and 1.5 g of rib from the infants and children were sampled for the stable isotope analysis. In the four cases in which these bones were not available to sample, cortical bone from the cranium, scapula, and pelvis was collected respectively (Table S1). RLK used a modified Longin method [107] to extract collagen from all of the bone samples at the University of Otago, Dunedin, New Zealand. Bone samples were cleaned with alum oxide air abrasive equipment (Bego Easyblast) and soaked in 0.5 M HCl at 4°C (changed every other day) until completely demineralized. The demineralized samples were then rinsed in deionized H_2_O until they reached a neutral pH. The samples were gelatanized at 70°C in a pH 3 solution for 48 hours, followed by filtering with 5–8 µm Ezee mesh filters (Elkay Laboratory Products) to remove any reflux-insoluble residues, and then ultrafiltered with Millipore Amicon Ultra-4 centrifugal filters (30,000 NMWL) to retain molecules larger than 30 kDa. The purified “collagen” was frozen and then lyophilized for 48 hours, and subsequently weighed into tin capsules before analysis by EA-IRMS at Iso-Analytical (Cheshire, UK). Analytical error was routinely ±0.1‰ for δ^13^C, ±0.2‰ for δ^15^N, and ±0.3‰ for δ^34^S (see Table S1).

Samples that did not meet the collagen quality criteria for carbon and nitrogen stable isotope ratios (%C over 30.0, %N above 11.0%, and atomic C:N ratio of 2.9–3.6) [108]–[110] and sulfur stable isotope ratios (an atomic C:S outside 600±300, an atomic N:S outside 200±100, and a wt %S above 60% for mammal bone) [111] were removed from the statistical analyses and interpretations. As ultrafilters substantially reduce the collagen yield (50% or more) [112], collagen yield was not used as a criteria for collagen quality in this study.

Strontium was purified from the enamel samples at the clean laboratory and MC-ICP-MS facility at the Department of Human Evolution, Max Planck Institute for Evolutionary Anthropology (Leipzig, Germany) under the supervision of MR and KJ using the ion exchange method outlined in Deniel and Pin [113]. Teeth were cleaned mechanically using a sonicated diamond saw attachment on a Dremmel drill to remove surface contaminants. A small piece of enamel was then cut from the tooth crown using a diamond edge saw attachment on a Dremmel drill. Using a magnifying lens and the Dremmel drill, the enamel was separated from any adjoining dentine and cleaned by ultrasonication in deionized water; the water was changed multiple times during this process. The enamel samples were transferred to the clean laboratory and dried thoroughly after being rinsed in ultrapure acetone. Samples weighing ∼10–20 mg were placed in separate Teflon beakers and digested at 120°C in 1 ml of 14.3 M HNO_3_. The solution containing the enamel sample was then evaporated on the hotplate until dry and subsequently mixed with 1 ml of 3 M HNO_3_. Clean, preconditioned 2 ml columns filled with clean Sr-spec resin (EiChrom, Darien, IL) were used to purify the strontium from the enamel solutions. Each sample was reloaded into its respective column three times. The resin containing the strontium was washed twice with 3 M HNO_3_, after which the strontium was eluted from the resin using ultrapure deionized water into a clean Teflon tube and evaporated on a hot plate until the sample was dry. Each sample was re-dissolved in 3% HNO_3_ and measured on a Thermo Fisher Neptune MC-ICP-MS instrument (Thermo Fisher Scientific, Dreieich, Germany) by KJ. The reference standard SRM 1486 and one beaker blank were measured in parallel for each run of twelve samples. Repeat measurements of the strontium isotope standard SRM 1486 yielded a mean (± SD) ^87^Sr/^86^Sr ratio of 0.70929±0.00003 (n = 7) and the results for the procedural blanks analyzed were negligible. To ensure the accuracy of the data, the international standard SRM 987 was measured, yielding a mean ^87^Sr/^86^Sr ratio of 0.71029±0.00001 (n = 48), and corrected to the accepted value of 0.71024±0.00004 [114], [115]. Strontium concentrations were estimated using a regression equation between known, stochiometrically determined concentrations (100, 400, and 700 ppb) and ^88^Sr signal intensities (V) measured for three SRM 987 standards and this method is described in full in Hartman and Richards [116].

## Results

### Modern samples for the dietary baseline

A full description of the stable isotope results of the modern dietary baseline are presented in Kinaston et al. [28].

### Prehistoric human and faunal carbon, nitrogen, and sulfur stable isotope ratios

All but three of the prehistoric humans (burials 2, 9, and 14) and one LL pig met the quality criteria for well-preserved collagen [108]–[110] and these four samples were removed from the following statistical analyses and interpretations. Overall, the average δ^13^C value (mean ± SD) for the Uripiv humans (n = 27) was –17.2‰ ±0.9‰ and ranged from –19.5‰ to –15.2‰. The mean human δ^15^N value was 9.5‰ ±1.4‰, ranging from 7.4‰ to 12.0‰. There were only enough adults in the PL period to assess the correlation between δ^13^C and δ^15^N values and this was not significant (n = 10, Spearman’s *r* = 0.338, *p = *0.340). The prehistoric pigs from Uripiv (n = 10) displayed an average δ^13^C value of –18.4‰ ±1.1‰, ranging from –20.1‰ to –16.9‰, and had an average δ^15^N value of 7.4‰ ±0.7‰, ranging from 5.9‰ to 8.6‰. Tables 1 and 2 detail the average δ^13^C and δ^15^N values as well as the ranges for the humans and pigs from Uripiv by temporal period (Lapita, LL, PL, and LPH) and age group (for the humans).

**Table 1:** Descriptive statistics for human bone collagen δ^13^C, δ^15^N, and δ^34^S values by age group and temporal period.

Period and age^a^	n =	δ^13^C (‰)	± SD	δ^15^N (‰)	± SD	δ^34^S n =	δ^34^S (‰)	± SD
Overall humans	27	−17.2	0.9	9.5	1.4	14	10.4	1.8
Lapita fetal/neonatal	4	−16.4	0.9	10.6	1.2	1	13.0	
Lapita ages 1.5–2.5years	2	−16.5	0.4	11.7	0.4			
LL adults	2	−17.6	0.4	8.3	0.2	2	11.1	0.6
LL 5-year-old child	1	−16.3		10.7				
PL adults	10	−17.0	0.4	9.0	0.6	6	10.8	1.0
PL 5-year-old child	1	−17.2		10.1		1	8.7	
PL child unknown age	1	−17.7		11.6				
PL ages 1.5–2.5years	2	−17.0	0.6	10.8	0.7	2	11.2	1.1
LPH adults	3	−19.2	0.4	7.8	0.6	2	7.1	1.1
LPH fetal/neonatal	1	−17.9		7.5				

a Later Lapita (LL), post-Lapita (PL), and late prehistoric/historic (LPH).

**Table 2:** Descriptive statistics for pig bone collagen δ^13^C, δ^15^N, and δ^34^S values for each temporal period.

Period^a^	n =	δ^13^C (‰)	± SD	δ^15^N (‰)	± SD	δ^34^S n =	δ^34^S (‰)	± SD
All	10	−18.4	1.1	7.4	0.7	4	11.5	1.8
Lapita	3	−18.3	1.3	7.5	1.0	1	12.8	
LL	3	−17.6	0.6	7.6	0.3	1	11.8	
PL	3	−18.8	0.8	6.9	0.9	2	10.6	2.6
LPH	1	−20.6		7.7				

a Later Lapita (LL), post-Lapita (PL), and late prehistoric/historic (LPH).

Sufficient collagen (∼10.0 mg) was extracted for sulfur stable isotope analysis from fourteen humans and four of the prehistoric pigs from Uripiv. All of the collagen samples analyzed for sulfur stable isotope ratios displayed atomic C:S, atomic N:S, and wt %S indicative of well-preserved collagen [111]. Overall, the humans displayed an average δ^34^S value of 10.4‰ ±1.8‰, ranging from 6.3‰ to 13.0‰. The pigs from Uripiv displayed an average δ^34^S value of 11.5‰ ±1.8‰, ranging from 8.8‰ to 12.8‰. The average δ^34^S values for the humans and pigs from each temporal period are also presented in Tables 1 and 2 respectively.

### Strontium isotope ratios

**Figure 2:**
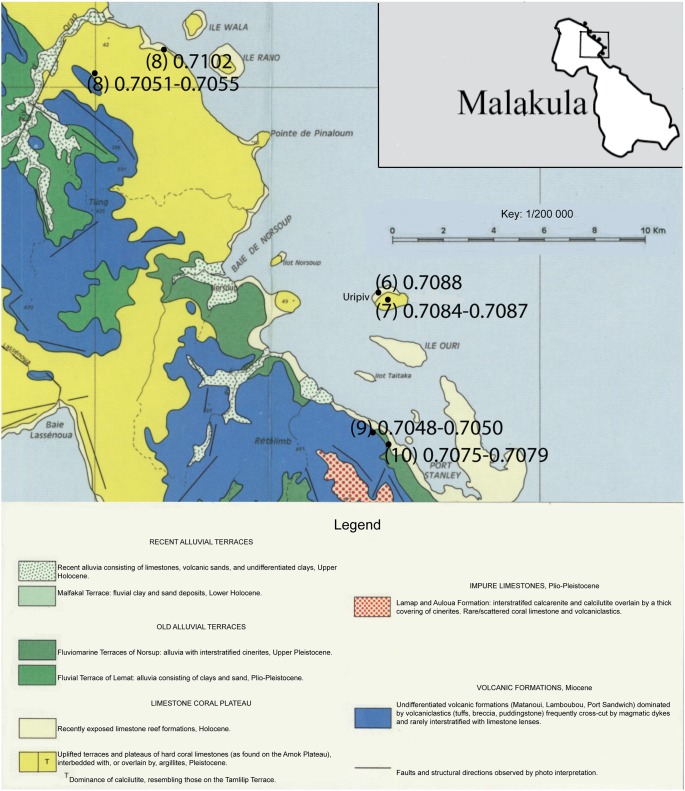
Geological map of northern Malakula with ranges of ^87^Sr/^86^Sr_plant_ ratios for each sampling location. © IRD, Quantin P (1972–1979) Nouvelles-Hébrides: Atlas des sols et de quelques données du milieu naturel. Bondy: Office de la Recherche Scientifique et Technique Outre-Mer.

**Figure 3:**
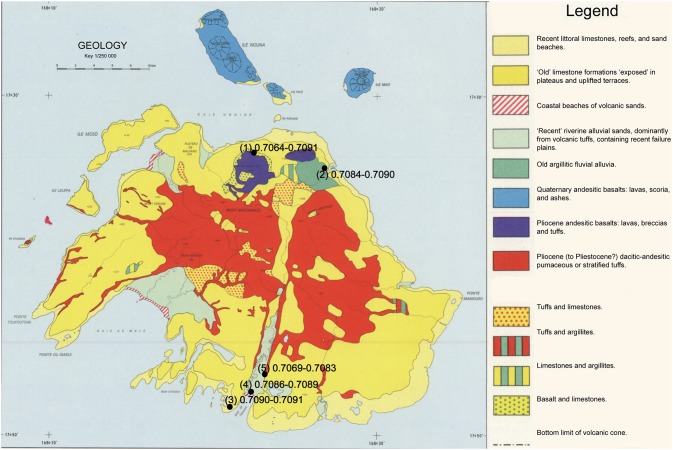
Geological map of Efate with ranges of ^87^Sr/^86^Sr_plant_ ratios for each sampling location. © IRD, Quantin P (1972–1979) Nouvelles-Hébrides: Atlas des sols et de quelques données du milieu naturel. Bondy: Office de la Recherche Scientifique et Technique Outre-Mer.

**Figure 4:**
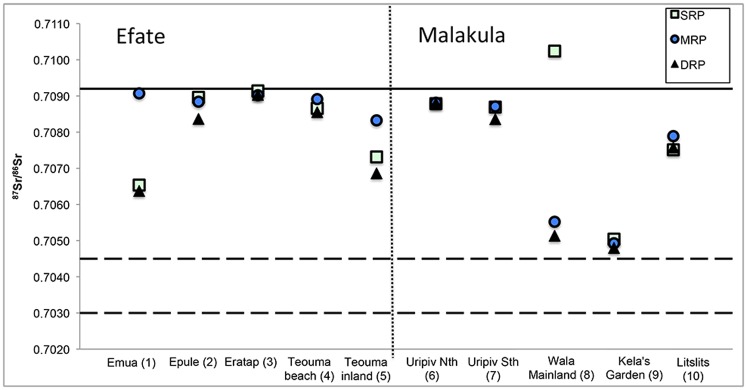
^87^Sr/^86^Sr_plant_ ratios of modern samples from Vanuatu. Symbols represent short-rooted plants (SRP), medium-rooted plants (MRP), and deep-rooted plants (DRP). The vertical dotted line delineates between sampling locations on Efate Island and Malakula. The solid horizontal line designates the average ^87^Sr/^86^Sr ratio for modern seawater and the two dashed lines represent the high and low ^87^Sr/^86^Sr ratios of oceanic basalt. The sampling location is detailed by the number in parentheses in Figures 2 and 3.

Data regarding plant species, ^87^Sr/^86^Sr_plant_ ratios, sampling location, and underlying geology are located in Figure 2, Figure 3, Figure 4, and Table S3. The average ^87^Sr/^86^Sr_plant_ (mean ± SD) ratio for the samples from Efate Island was 0.7083±0.0010 (range of 0.7064 to 0.7091). The average ^87^Sr/^86^Sr_plant_ ratio of the samples from Northeast Malakula, including Uripiv, was 0.7074±0.0018 (range of 0.7102 to 0.7048). The samples analyzed from Uripiv displayed relatively homogeneous ^87^Sr/^86^Sr_plant_ ratios, averaging 0.7087±0.0002 (range of 0.7084 to 0.7088). The plants collected from the northeast coast of the Malakula Mainland displayed an average of 0.7065±0.0019 and, with the exception of one short-rooted plant from a seaside garden (^87^Sr/^86^Sr_plant_ ratio of 0.7102), displayed less radiogenic and more variable ^87^Sr/^86^Sr_plant_ ratios compared with the Uripiv plants (Fig. 4).

All fifteen teeth yielded enamel strontium isotope ratios, which are presented alongside the strontium concentrations in Table S1. Overall, the human enamel samples (n = 15) displayed an average ^87^Sr/^86^Sr_enamel_ ratio of 0.7084±0.0003 (range of 0.7076 to 0.7088), and the average ^87^Sr/^86^Sr_enamel_ ratio and range of ^87^Sr/^86^Sr_enamel_ ratios for the individuals from each period are presented in Table 3.

**Table 3:** Descriptive statistics for ^87^Sr/^86^Sr_enamel_ ratios of the Uripiv humans.

Period^a^	n =	^87^Sr/^86^Sr	± SD	Max	Min
All	15	0.7084	0.0003	0.7076	0.7088
Lapita	1	0.7087			
LL	2	0.7084	0	0.7084	0.7084
PL	11	0.7084	0.0004	0.7076	0.7088
LPH	1	0.7086			

a Lapita, later Lapita (LL), post-Lapita (PL), and late prehistoric/Historic (LPH) periods.

## Discussion

The isotope results for the humans and pigs from Uripiv are discussed in reference to the aims presented in the introduction.

### Human and pig diets

**Figure 5:**
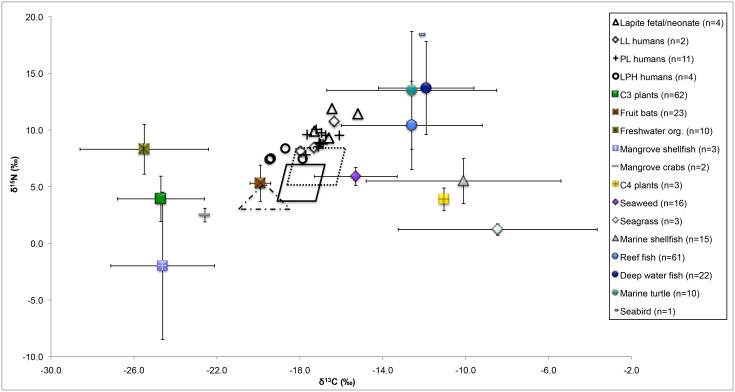
Uripiv human δ^13^C and δ^15^N values in relation to a tropical Pacific island dietary baseline. Only adults, children aged 5 years and above, and fetal/neonatal individuals shown on the graph. The dotted, solid, and dashed shapes delineate the potential protein diet of the Lapita (far right, dotted), PL and LL (center, solid), and LPH (left, dashed) individuals after correcting for trophic level differences (4.0‰ for δ^15^N values and 1.0‰ for δ^13^C values). Note that the modern δ^13^C values have been corrected for the Suess effect.

The first aim was to use stable isotope analysis to assess if there was a transition to more plant-based foods from the Lapita to the PL and LPH periods for both humans and pigs. Admittedly, the age distribution between the temporal periods is disparate and the sample sizes from the Lapita and LPH periods are small. However the intact burials from Uripiv constitute one of the largest skeletal assemblages in the Pacific Islands that dates to these periods and therefore provides a unique opportunity to assess diet, subsistence practices, and animal husbandry methods. The average δ^13^C value of the modern and prehistoric fruit bat bones (−19.9‰ ±0.5) can be used as a proxy for a purely terrestrial C_3_ plant-based diet. This value corresponds with the δ^13^C value of –20.0‰±1.0‰ that other studies have reported as representing a purely C_3_-based terrestrial diet in the Pacific [117], [118]. If a trophic effect of 1–2‰ for δ^13^C values and 3–5‰ for δ^15^N values is taken into account, it can be established from the dietary baseline that the marine protein resources for humans and pigs from all periods on Uripiv were lower trophic level organisms likely from the inshore environment, seagrass meadows, and fringing reef, but may have included smaller proportions of pelagic fish, especially those with low δ^15^N values similar to the modern tuna analyzed in Vanuatu (Fig. 5). Mangrove organisms may also have been on the menu on Uripiv, but they are difficult to discern as they display the δ^13^C values of C_3_ terrestrial plants and the δ^15^N values of low trophic level organisms. A similar broad-spectrum approach to foraging nearby reef and inshore environments was found at other Lapita sites and is thought to be an efficient way to maximize food yields while providing a wide range of nutrients [119], [120].

**Figure 6:**
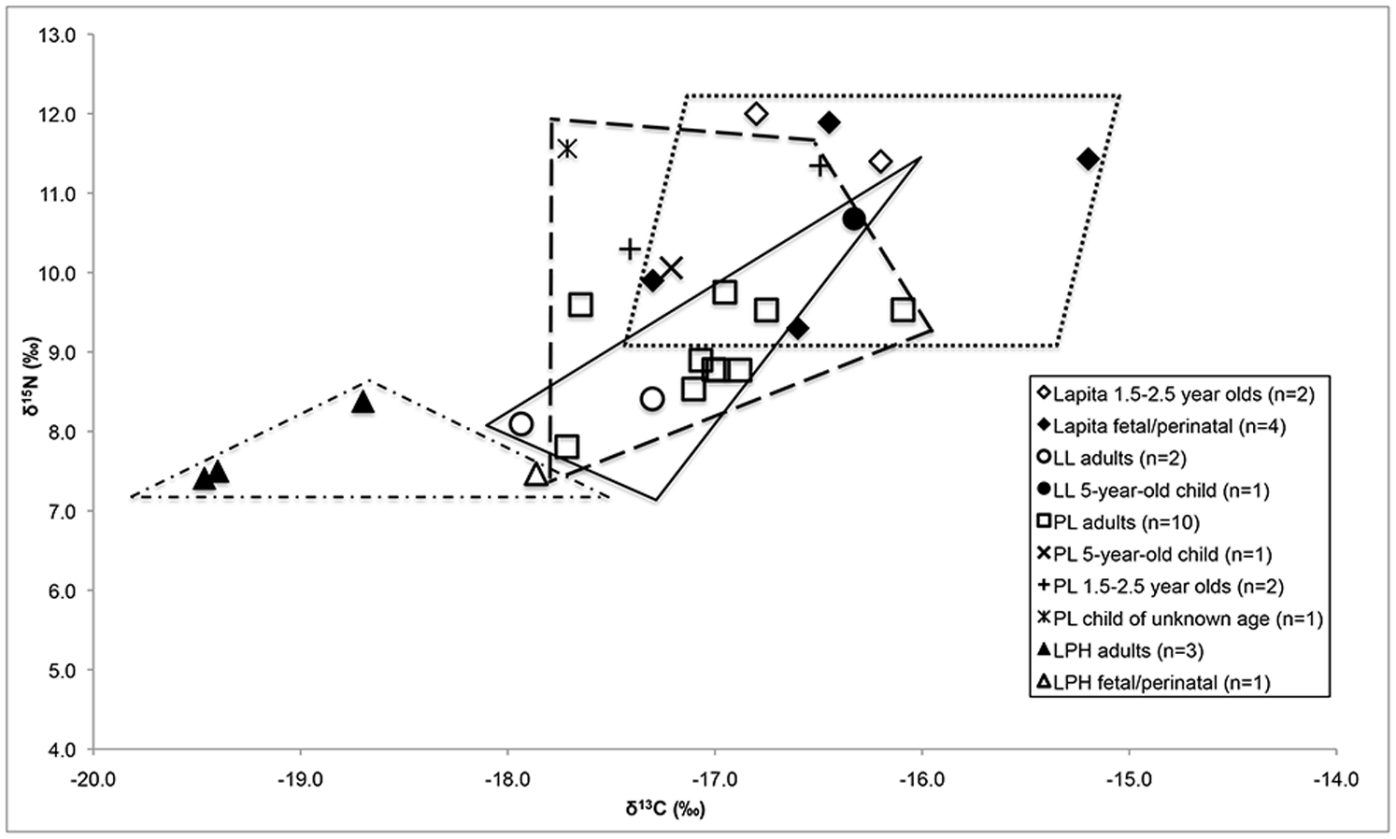
Comparison of Uripiv human δ^13^C and δ^15^N values from individuals of all ages. The dotted line delineates the Lapita humans (right), the solid line delineates the LL humans (center), the dashed line delineates the PL humans (center), and the dashes with dots delineate the LPH humans (left).

The major human dietary differences between the temporal periods were observed in the δ^13^C values. If we assume that the fetal and perinatal individuals from the earliest Lapita layer (n = 4) on Uripiv are representative of their maternal δ^13^C and δ^15^N values, we can infer that the diet during the initial occupation of the island included proportionally more marine food than the diet during the LL period, as the δ^13^C and δ^15^N values of the Lapita individuals are 1.2‰ and 2.4‰ higher than those of the adults of the LL period (Fig. 6). This trend is not surprising as it is to be expected that the earliest inhabitants utilized the most easily accessible marine resources when they were first establishing crops, a pattern also observed in palaeodietary studies of an early Lapita (ca. 3000 BP) cemetery sample from the site of Teouma, Efate Island, Vanuatu [28], [29]. Individuals of the earlier Lapita period at Uripiv who died between 1.5 and 2.5 years of age displayed similar δ^13^C values (diff 0.1‰) but higher δ^15^N values (1.1‰) compared with the fetal/perinatal individuals of the same period. The higher δ^15^N values likely reflect the fact that these individuals were breastfeeding at the time of death, but the size of the samples and lack of adult females from this period make more definitive conclusions difficult [71], [121].

The PL adult individuals (n = 10) displayed slightly lower δ^13^C values (diff 0.6‰) and slightly higher δ^15^N values (diff 0.7‰) compared with the LL adult individuals (n = 2). Although there was a decrease in δ^34^S values from the LL period to the PL period, the sample sizes are small and the difference between the LL and PL periods is only 0.3‰. The similarity in δ^13^C, δ^15^N, and δ^34^S values between the adults of the LL and PL periods likely reflects the fact that people had established horticulture during both these periods and, in addition to these plant products, were subsisting on low trophic level organisms from the rocky shore, reef, seagrass meadows, and mangroves. A marine focus was also suggested from the moderate size of stone ovens documented on Uripiv during both the Lapita and PL periods, which were likely more suited for cooking a variety of food resources, rather than large amounts of farinaceous plant foods [122].

Interestingly, the one five-year-old child of the LL period displayed higher δ^13^C (diff 1.3‰) and δ^15^N values (diff 2.5‰) compared with the adults of this period, suggesting this child ate more marine protein. The two children of the PL period (one 5 years old and one of unknown age) displayed lower δ^13^C values (diff 0.2‰ and 0.7‰ respectively) but higher δ^15^N values (diff 1.1‰ and 2.6‰ respectively) compared with the adults of the same period, indicating that, like the LL child, they were likely eating more marine and mangrove organisms than the adults. Although the sample sizes are small, this trend may suggest that children were collecting and eating marine organisms as snacks, a trend observed across many Pacific islands today [123]–[125]. The child of unknown age may be displaying a breastfeeding signal as the PL individuals aged between 1.5 and 2.5 years at the time of death (n = 2) also displayed high δ^15^N values compared with the adults of the same period (diff 1.8‰), but no differences were observed between their δ^13^C values. PL males (n = 2) displayed slightly higher δ^13^C and δ^15^N values compared with the females (n = 4) of the same period (diff 0.6‰ and 0.5‰ respectively), possibly indicating a higher consumption of marine foods by the males, but the samples sizes and differences are too small to further analyze possible sexual differences in diet.

**Figure 7:**
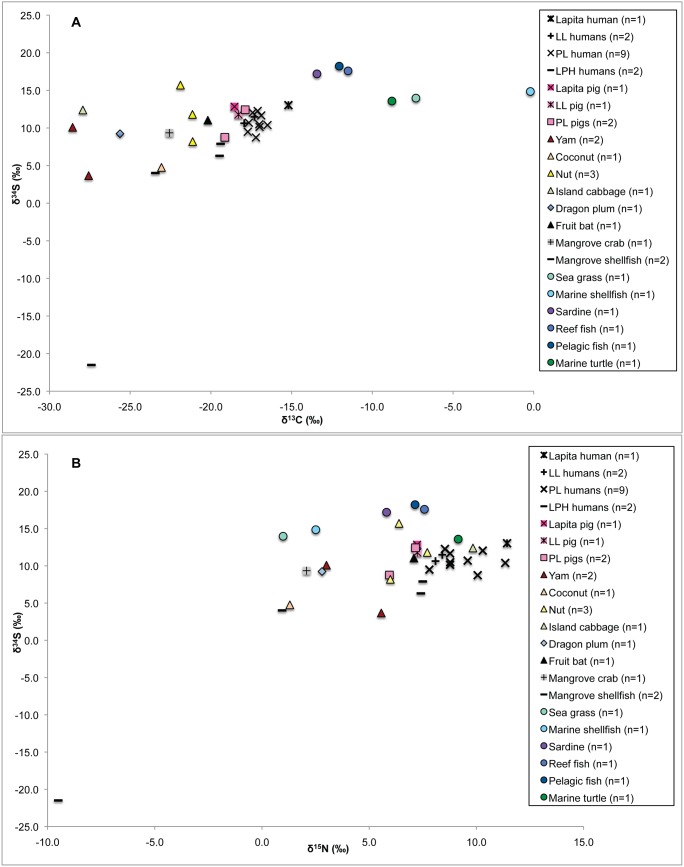
Uripiv human and pig δ^13^C and δ^34^S (A) and δ^15^N and δ^34^S (B) values. Human values plotted in relation to modern plant and animals from Vanuatu. Note that the modern δ^13^C values have been corrected for the Suess effect.

There was a substantial difference in the δ^13^C, δ^15^N, and δ^34^S values amongst the adults from the PL (n = 10) and LPH periods (n = 3) (diff 2.2 ‰ for δ^13^C values; 1.2 ‰ for δ^15^N values; 3.7‰ for δ^34^S values) (Fig. 7), likely indicating a heavier reliance on C_3_ horticultural and arboricultural plant foods during the later period. The low δ^34^S values during the LPH period may indicate that people were utilizing garden land adjacent to the Malakula mainland that was farther away from the coast and thus less affected by sea spray. The apparent intensive horticultural/arboricultural practices utilized during the LPH period may have been facilitated by the deposition of fertile volcanic tephra over the landscape from eruptions of the Ambrym volcano ca. 1800 BP, which continued periodically through to the modern period, and, possibly, the Kuwae volcano ca. 600 BP [44]. The one LPH fetal individual displayed lower δ^13^C values (1.3‰) and slightly higher δ^15^N values (0.3‰) than the adult females of the same temporal period (n = 3). The stable isotope results of the infants from Uripiv should be interpreted with caution as the fetal and perinatal individuals from Teouma displayed significantly lower δ^13^C values and significantly higher δ^15^N values compared with the mean female values in that cemetery sample, possibly as a result of in utero stress due to maternal ill-health [36].

It must also be noted that the sulfur stable isotope results are relatively ambiguous with regard to interpretations of terrestrial and marine protein consumption at the site. Based on the high δ^34^S values of the terrestrial organisms analyzed for the dietary baseline, it can be suggested that there is a considerable amount of marine-derived sulfur in modern coastal terrestrial foodwebs as a result of the sea-spray effect. Active volcanism during the entire period of human settlement on Vanuatu may also have contributed substantial amounts of sulfur to the environment through the deposition of sulfurous ‘vog’ (volcanic gasses that react with oxygen, moisture, and sunlight) and ash. Although there were observable differences between the PL and LPH periods, which may be related to changes in garden location, the interpretation of palaeodiet from δ^34^S values is difficult in coastal, volcanic, tropical Pacific islands [28], [30], [77].

There are few differences in the average pig δ^13^C and δ^15^N values amongst the Lapita, LL and PL pigs, indicating that they were fed similar amounts of C_3_ terrestrial plants over time at the site. However, the Lapita pigs displayed a wider range of δ^13^C and δ^15^N values (n = 3, –19.4‰ to –16.9‰ and 6.7‰ to 8.6‰) compared to the LL pigs (n = 3, –18. 3‰ to –17.3‰ and 7.2‰ to 7.8‰) but were comparable to the range in δ^13^C and δ^15^N values of the PL pigs (n = 3, –19.3‰ to –17.9‰ and 5.9‰ to 7.7‰). Although the sample sizes are small, the range of δ^13^C and δ^15^N values indicates more variation in husbandry practices during the earliest Lapita and PL periods, either on Uripiv or as a result of transporting pigs to the island as a trade item. The single LPH pig displayed a δ^13^C value of –20.6‰ and a δ^15^N value of 7.7‰, indicating this pig subsisted entirely on terrestrial resources, some of which may have come from mangrove environments. With the exception of the LL period, during which pigs and humans displayed identical mean δ^13^C values, the pigs displayed lower δ^13^C and δ^15^N values when compared with the humans of the same period, suggesting they were fed less marine foods and more horticultural produce than their human counterparts. It is possible that some of these animals may have been brought to the island from other areas. A strontium isotope analysis of Lapita pigs from Watom Island in the Bismarck Archipelago in New Guinea found that some pigs were likely raised elsewhere and imported [16], and historical and modern ethnographic accounts detail the trade of pigs as prestige items throughout the Pacific Islands [126], [127]. The exchange of pigs in relation to grade taking ceremonies in Malakula is also well documented in the early historic period [128], [129].

A heavy reliance on horticultural and arboricultural foods can be observed in the LPH period on Uripiv, during which both people and pigs were subsisting heavily on terrestrial plant products. Food consumption patterns today mirror this scenario on Malakula and across the Pacific, where starchy vegetable staples constitute at least 80% of the foods eaten, and all other foods are considered supplementary (e.g. meat, fish, shellfish, and greens) or snacks (e.g. nuts and fruit) [128], [130]–[132].

**Figure 8:**
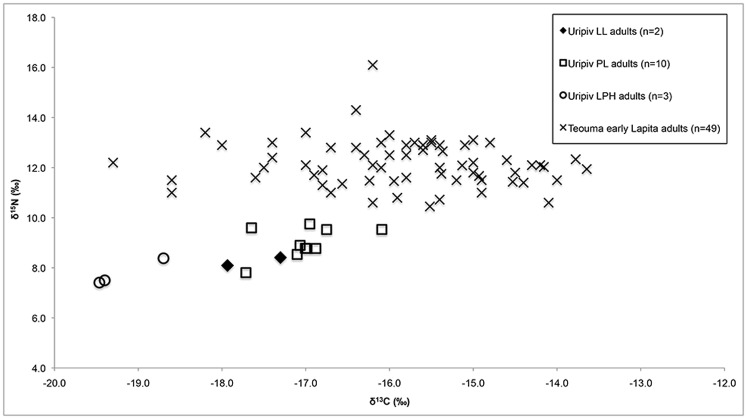
Comparison of bone collagen δ^13^C and δ^15^N values of Uripiv and Teouma adult humans.

In comparison, a previous stable isotope analysis has suggested that the protein diet of the people of the early Lapita site of Teouma, Vanuatu (ca. 3000 BP, n = 49) was representative of the consumption of inshore and reef organisms, native terrestrial animals, and domestic animals [28], [29]. It is difficult to directly compare the stable isotope ratios of Lapita individuals from Uripiv and Teouma due to the large proportion of young subadult individuals in the Uripiv sample. However, the LL, PL, and LPH adults from Uripiv displayed lower δ^13^C values (by 1.9‰, 1.4‰ and 3.5‰ respectively) and δ^15^N values (by 3.9‰, 3.1‰ and 4.3‰ respectively) compared with the adult individuals from Teouma (Fig. 8). Based on these results it can be suggested that the diet on Uripiv during all settlement periods was more terrestrial and from lower trophic levels (most likely horticultural products) compared with the early Lapita community to the south, who are thought to have been amongst the first inhabitants of Efate, Vanuatu. The isotope data thus support the archaeological models of initial broad-spectrum resource gathering with some horticulture and animal husbandry during the earliest Lapita settlement of Vanuatu, followed by the intensification of gardening and animal husbandry in later periods, especially after local marine and terrestrial resources were depleted.

**Figure 9:**
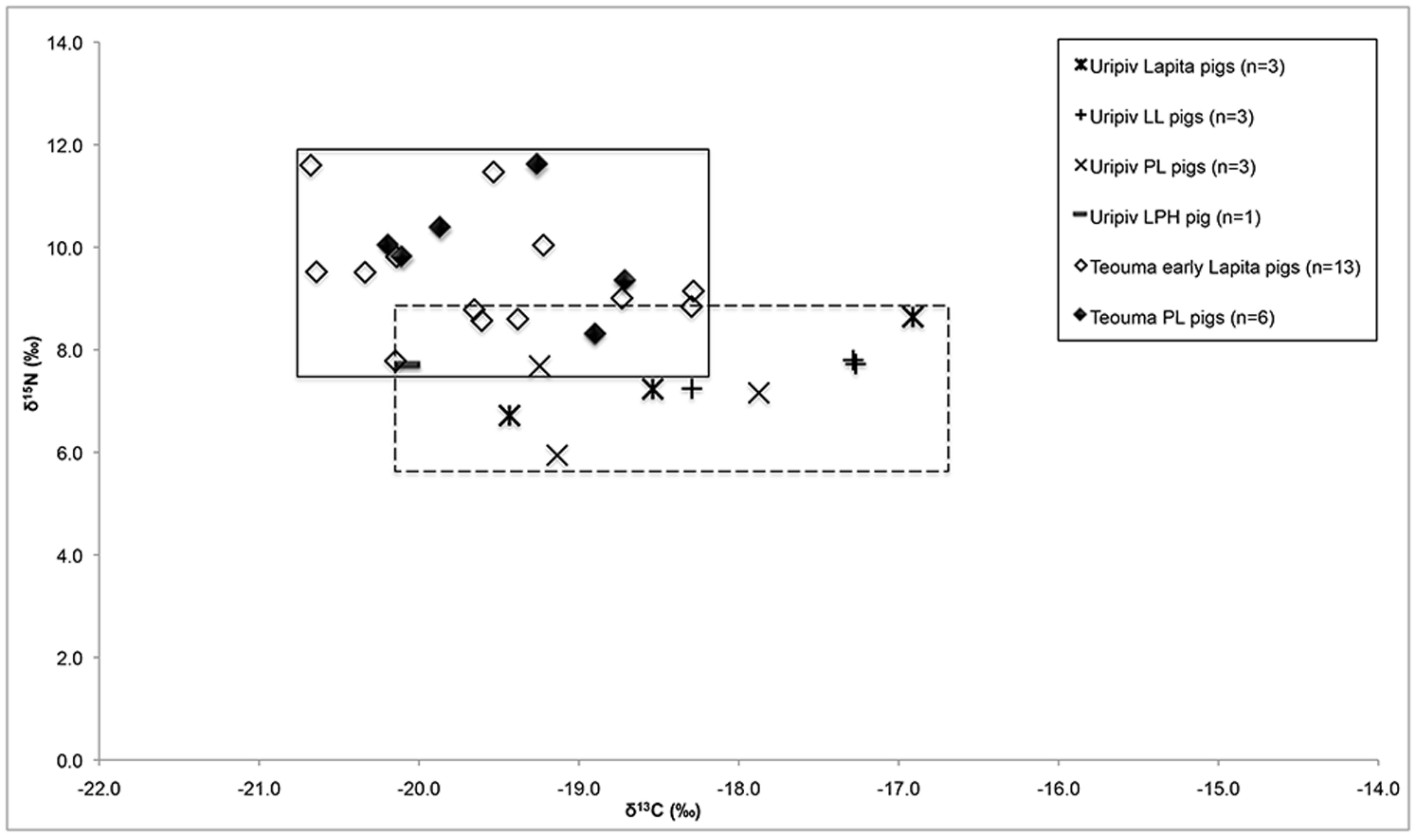
Comparison of bone collagen ^13^C and δ^15^N values of Uripiv and Teouma pigs.

Interestingly, the diets of the early Lapita and PL pigs from Teouma were more terrestrial and from higher trophic levels compared with the pigs from Uripiv (Fig. 9). This indicates that animal husbandry practices differed between the communities. It has been suggested that the pigs at Teouma were foraging for wild foods, including insects and human feces [28], which supports the premise of a less intensive feeding management strategy on the large island of Efate, and is similar to that suggested by Clark et al. [133] for pigs in Palau, Micronesia. The low trophic level, mostly terrestrial diet of the Uripiv pigs likely consisted of a large proportion of starchy root vegetables, indicating that there was a more controlled method of animal husbandry on Uripiv (or nearby islands if the pigs were imported) throughout the prehistoric period compared with the early Lapita settlement of Teouma. The stable isotope evidence suggests that pigs on Uripiv during the LPH period were primarily fed human food scraps and horticultural plant foods, with little or no opportunity to root for wild protein sources. A highly controlled method of pig husbandry is common throughout modern Pacific communities, especially in those with restricted land areas such as Uripiv [134]. Today on Uripiv, pigs are fed a large proportion of plant foods from gardens in addition to coconuts, and are penned or tethered for most of their lives to avoid destruction to gardens - a practice that is likely reflected in the stable isotope values of the prehistoric pigs from the island.

### Human mobility

The second aim of this study was to use strontium isotope ratios to ascertain if human mobility could be identified at the site and if there were observable changes in mobility patterns between the Lapita and later periods. In order to interpret the human strontium isotope ratios, it was necessary to establish the expected labile ^87^Sr/^86^Sr ratios for Uripiv and the surrounding areas. The baseline strontium mapping conducted in this study indicated that, in some areas, there was considerable variability in soil ^87^Sr/^86^Sr ratios from a single location, which was likely associated with soil depth. In all the areas sampled, the deep-rooted plants displayed the least radiogenic ^87^Sr/^86^Sr_plant_ ratios compared with medium and short-rooted plants from the same sampling localities (Fig. 4). A likely reason for this is that the topsoil ^87^Sr/^86^Sr ratios, especially in coastal locations, are more radiogenic as a result of marine-derived precipitation or sea spray mixing with the upper soil, which is a phenomenon also observed in the northern hemisphere [90]. This sea spray effect may influence the ^87^Sr/^86^Sr ratios of coastal horticultural plants from the Pacific, as they are relatively shallow-rooted species such as yam, taro (*Colocasia* spp. and non-*Colocasia* spp.), and banana.

Regardless of the effects of sea spray, the average ^87^Sr/^86^Sr_plant_ ratios show that there are observable differences between regions in Vanuatu as a result of variation in the underlying geology. With the exception of one outlier sample from a short-rooted plant from a seaside garden, the ^87^Sr/^86^Sr_plant_ ratios of the Malakula mainland were much less radiogenic than those from the limestone island of Uripiv. The ^87^Sr/^86^Sr_plant_ ratios observed on Uripiv were less radiogenic than the limestone geological base rock value, which should reflect the ^87^Sr/^86^Sr ratio of seawater (0.7092). A likely explanation for this is that the island’s soil has developed from a combination of the weathering of the underlying limestone, the deposition of volcanic tephra, and the decomposition of plant matter.

**Figure 10:**
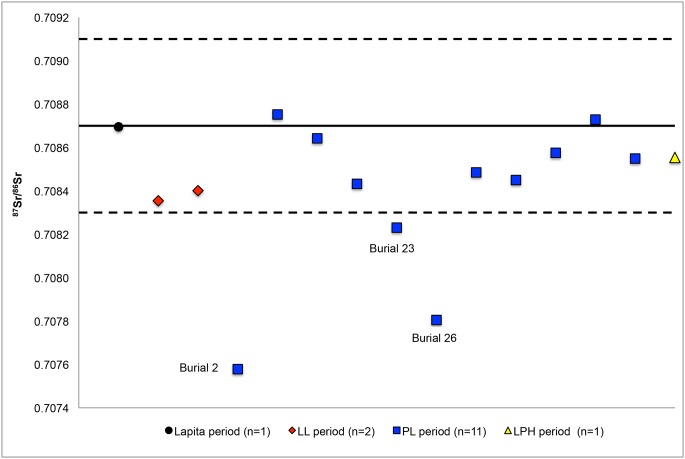
Human ^87^Sr/^86^Sr_enamel_ ratios plotted in relation to the biologically available average ^87^Sr/^86^Sr_plant_ ratio of Uripiv. The solid line represents the average ^87^Sr/^86^Sr_plant_ ratio for Uripiv and the dashed lines delineate±2 SD from this mean.

Twelve of the fifteen individuals from Uripiv displayed ^87^Sr/^86^Sr_enamel_ ratios that fell within the average bioavailable ^87^Sr/^86^Sr_plant_ ratio (0.7087±0.002) for Uripiv (Fig. 10). The Lapita individual, both the LL individuals, eight of the PL individuals, and the one LPH individual were considered ‘local’. From this we can suggest that most of these individuals likely spent their childhoods on Uripiv, but may have also lived on the nearby offshore islands of Vao, Rano, Atchin, and Wala, which – like Uripiv-are limestone-based and likely display analogous labile ^87^Sr/^86^Sr ratios. The analyses of the ^87^Sr/^86^Sr ratios of the deciduous teeth from burial 1 (a Lapita 1.5-year-old), burial 8 (a LL 5-year-old), burial 27 (a PL 1.8–2.5-year-old), and burial 31 (a PL 2.5-year-old) indicate that their mothers’ spent time on Uripiv or on nearby offshore islands with similar labile ^87^Sr/^86^Sr ratios while these infants’ were in utero or later, when they were breastfeeding.

All three of the non-local individuals were of the PL period. Permanent teeth were sampled from two of these individuals (burial 2, a female, and burial 23, a male) and the observed ^87^Sr/^86^Sr_enamel_ ratios are a reflection of the food sources eaten by the individuals during childhood. A deciduous second molar was sampled from burial 26 (a 5-year-old child), and because the crown of this tooth is complete at about one year of age, the ^87^Sr/^86^Sr_enamel_ ratios are likely representative of the mother’s dietary and, to a lesser degree, skeletal, ^87^Sr/^86^Sr ratios during pregnancy and breastfeeding, in addition to the source of the supplementary foods eaten by the infant. This suggests that the mother was living elsewhere when the child was at least one year of age, after which time the child moved to Uripiv sometime before his or her death around the age of five. All three non-local individuals displayed ^87^Sr/^86^Sr_enamel_ ratios that were less radiogenic than the mean ^87^Sr/^86^Sr_plant_ ratio of Uripiv, suggesting that they may have spent their childhoods on the Malakula mainland or another area of Vanuatu with a similar underlying geology. It is difficult to pinpoint exact origins, especially in the case of Vanuatu, as the range in values will be between that of oceanic basalt (0.7030–0.7045) and seawater (0.7092).

The mobility observed in the PL period may be a result of increased population sizes and the establishment of settlements on the Malakula mainland, and the subsequent movement of people between these areas, possibly for marriage. Unfortunately, due to the small sample sizes it is difficult to meaningfully interpret patterns of migration related to sex within the PL period or possible changes in migration patterns between the Lapita and PL periods.

## Conclusions

Bedford ([41]:156) has suggested that “on initial arrival on an island it can be expected that there is a short period, before the establishment of agricultural systems, when populations were heavily reliant on readily procurable local marine and terrestrial resources”. The stable isotope results from Uripiv support this model and suggest a more marine-focused subsistence strategy during the initial Lapita occupation of the island. As local resources were likely depleted, there was a transition to a greater reliance on cultivated plants during the later Lapita and post-Lapita periods, as horticulture and arboriculture were intensified and adapted to local conditions. The method of pig husbandry appears to have been similar during both the Lapita and post-Lapita periods, but the variation in stable isotope values of the pigs may suggest their importation from other areas. The analysis of a larger sample and, possibly, the analysis of strontium isotopes in pig tooth enamel, will be necessary to fully interpret these results. During the late prehistoric/historic period, it is clear that people and pigs were relying much more heavily on terrestrial plants than at any previous time, likely mirroring traditional subsistence practices observed in Vanuatu today [132]. The strontium isotope data indicate some level of mobility in the post-Lapita period, with some people moving to Uripiv after childhood, possibly with young infants. This pattern could be indicative of marriage patterns or relocation for other means. Without a larger sample from the Lapita period it is difficult to make assumptions regarding changes in the level of mobility over time at the site. However, the presence of a number of non-local individuals at the site during the post-Lapita period does support the archaeological evidence that there was a high level of mobility occurring on a more regionalized scale at this time. It would be beneficial for future studies to focus on establishing a more comprehensive strontium isotope baseline for Vanuatu using a wider range of materials such as groundwater, soil leachate, rocks, and short ranging animals.

## Supporting Information

Table S1 **Temporal information, demographic data, bone collagen δ^13^C, δ^15^N, and δ^34^S values, collagen quality indicators, ^87^Sr/^86^Sr_enamel_ ratios, and strontium concentrations (Sr) for the humans from Uripiv.**
(DOCX)

Table S2 **Temporal period, bone collagen δ^13^C, δ^15^N, and δ^34^S values, and collagen quality indicators of the prehistoric pigs (** ***Sus scrofa*** **) from Uripiv.**
(DOCX)

Table S3 **Plant type, sampling location, species, ^87^Sr/^86^Sr_plant_ ratios, and strontium concentration (Sr) of the modern plants analysed in this study.**
(DOCX)
