# Supplementary material for: Diet and Human Mobility from the Lapita to the Early Historic Period on Uripiv Island, Northeast Malakula, Vanuatu
Source: PLoS One. 2014 Aug 20;9(8):e104071. doi: 10.1371/journal.pone.0104071 (PMC4139273; doi:10.1371/journal.pone.0104071)
Supplement: Table S1 — Temporal information, demographic data, bone collagen δ13C, δ15N, and δ34S values, collagen quality indicators, 87Sr/86Srenamel ratios, and strontium concentrations (Sr) for the humans from Uripiv. (DOCX) [file pone.0104071.s001.docx]

Table S1. Temporal information, demographic data, bone collagen δ^13^C, δ^15^N, and δ^34^S values, collagen quality indicators, ^87^Sr/^86^Sr_enamel_ ratios, and strontium concentrations (Sr) for the humans from Uripiv.

| Burial | Period^a^ | AMS date^b^ | Element^c^ | Sex^d^ | Age^e^ | %N^fg^ | δ^15^N (‰) | %C | δ^13^C (‰) | C:N | %S^h^ | δ^34^S (‰) | N:S | C:S | Tooth^i^ | CM age (yrs)^j^ | S-EVA ID^k^ | Enamel ^87^Sr/^86^Sr | Sr (ppm) |
| --- | --- | --- | --- | --- | --- | --- | --- | --- | --- | --- | --- | --- | --- | --- | --- | --- | --- | --- | --- |
| UH1 | Lapita |  | Ribs | SA | 1.5 yrs | 15.1 | 12.0 | 42.2 | -16.8 | 3.3 |  |  |  |  | 84 | 0.3 - 0.8 | 23721 | 0.70870 | 497.7 |
| UH2 | PL |  | LB shaft | F | Adult | ***4.1*** | ***2.7*** | ***9.7*** | ***-21.7*** | ***2.7*** |  |  |  |  | 25 | 6.5 - 6.6 | 23722 | 0.70758 | 293.8 |
| UH5 | Lapita |  | Ribs | SA | 33 fwks | 14.4 | 9.9 | 38.9 | -17.3 | 3.1 |  |  |  |  |  |  |  |  |  |
| UH6 | Lapita |  | Ribs | SA | 34 fwks | 14.7 | 9.3 | 41.0 | -16.6 | 3.3 |  |  |  |  |  |  |  |  |  |
| UH7 | Lapita |  | Ribs | SA | 1.5 yrs | 14.4 | 11.4 | 38.7 | -16.2 | 3.1 |  |  |  |  |  |  |  |  |  |
| UH8 | LL | 2515 ± 28 | Ribs | SA | 5 yrs | 14.5 | 10.7 | 44.0 | -16.3 | 3.5 |  |  |  |  | 52 | 0.2 - 0.4 | 23723 | 0.70835 | 242.2 |
| UH9 | PL |  | LB shaft | UK | Adult | ***8.5*** | ***8.8*** | ***21.5*** | ***-18.2*** | ***2.9*** |  |  |  |  |  |  |  |  |  |
| UH10 | LPH | 185 ± 30 | Ribs | SA | 37 fwks | 14.5 | 7.5 | 41.9 | -17.9 | 3.4 |  |  |  |  |  |  |  |  |  |
| UH11 | PL | 2030 ± 30 | Ulna shaft | UK | Adult | 14.5 | 9.6 | 41.0 | -17.6 | 3.3 | 0.21 | 10.7 | 155.1 | 509.7 |  |  |  |  |  |
| UH12 | PL |  | Crania | F? | Adult | 14.3 | 7.8 | 39.8 | -17.7 | 3.3 | 0.20 | 9.5 | 160.4 | 521.9 |  |  |  |  |  |
| UH14 | PL | 2339 ± 30 | LB shaft | UK | Adult | ***13.5*** | ***9.0*** | ***42.8*** | ***-17.4*** | ***3.7*** |  |  |  |  |  |  |  |  |  |
| UH15 | Lapita | 2608 ± 30 | Ribs | SA | 38 fwks | 14.7 | 11.4 | 41.6 | -15.2 | 3.3 | 0.22 | 13.0 | 155.5 | 513.9 |  |  |  |  |  |
| UH16 | PL | 2440 ± 30 | Ulna shaft | F? | Adult | 16.2 | 8.5 | 46.0 | -17.1 | 3.3 | 0.23 | 12.3 | 160.5 | 532.7 | 17 | 6.6 - 6.8 | 23724 | 0.70875 | 437.6 |
| UH17 | PL | 2268 ± 30 | Crania | M? | Adult | 15.7 | 8.8 | 44.0 | -16.9 | 3.3 | 0.21 | 11.7 | 170.8 | 559.9 | 16 | 2.4 - 2.5 | 23725 | 0.70864 | 253.2 |
| UH18 | PL | 2111 ± 30 | Femur shaft | F? | Adult | 15.4 | 8.8 | 43.9 | -17.0 | 3.3 | 0.19 | 10.5 | 184.7 | 616.8 | 17 | 6.6 - 6.8 | 23726 | 0.70843 | 29.1 |
| UH19 | LL | 2530 ± 28 | Tibia shaft | F | Adult | 15.6 | 8.4 | 45.2 | -17.3 | 3.4 | 0.20 | 11.5 | 176.6 | 597.5 | 16 | 2.4 - 2.5 | 23727 | 0.70840 | 240.3 |
| UH20 | PL |  | Pelvis | UK | Adult | 14.3 | 9.8 | 41.9 | -17.0 | 3.4 |  |  |  |  |  |  |  |  |  |
| UH23 | PL | 2310 ± 33 | LB shaft | M | Adult | 15.2 | 9.5 | 44.9 | -16.1 | 3.4 |  |  |  |  | 37 | 6.6 - 6.8 | 23728 | 0.70823 | 326.7 |
| UH25 | PL |  | Tibia shaft | SA | UKC | 13.5 | 11.6 | 39.6 | -17.7 | 3.4 |  |  |  |  |  |  |  |  |  |
| UH26 | PL |  | Humerus shaft | SA | 5 yrs | 15.5 | 10.1 | 44.8 | -17.2 | 3.4 | 0.18 | 8.7 | 196.2 | 663.2 | 85 | 0.7 - 1.4 | 23698 | 0.70780 | 210.6 |
| UH27 | PL | 2294 ± 32 | Ribs | SA | 1.8-2.5 yrs | 15.1 | 11.3 | 45.0 | -16.5 | 3.5 | 0.19 | 10.4 | 182.0 | 631.6 | 85 | 0.7 - 1.4 | 23729 | 0.70849 | 322.0 |
| UH28 | Lapita |  | Ribs | SA | 40 fwks | 15.0 | 11.9 | 45.1 | -16.4 | 3.5 |  |  |  |  |  |  |  |  |  |
| UH29 | LPH | 247 ± 25 | LB shaft | F | Adult | 16.0 | 7.4 | 44.3 | -19.5 | 3.2 | 0.23 | 6.3 | 159.7 | 515.1 | 17 | 6.6 - 6.8 | 23730 | 0.70855 | 315.4 |
| UH30 | LPH |  | LB shaft | F | Adult | 15.5 | 8.4 | 45.0 | -18.7 | 3.4 |  |  |  |  |  |  |  |  |  |
| UH31 | PL |  | Ribs | SA | 2.5 yrs | 15.2 | 10.3 | 43.3 | -17.4 | 3.3 | 0.24 | 12.0 | 146.9 | 490.0 | 84 | 0.3 - 0.8 | 23731 | 0.70845 | 243.8 |
| UH32 | PL |  | LB shaft | F | Adult | 13.8 | 9.5 | 40.6 | -16.8 | 3.4 |  |  |  |  | 47 | 6.6 - 6.8 | 23732 | 0.70858 | 221.2 |
| UH33 | PL | 2101 ± 25 | Scapula | UK | Adult | 15.5 | 8.9 | 44.6 | -17.1 | 3.4 |  |  |  |  | 48 | 12.4 - 12.6 | 23733 | 0.70873 | 357.1 |
| UH34 | LPH | 151 ± 25 | LB shaft | F? | Adult | 15.1 | 7.5 | 42.3 | -19.4 | 3.3 | 0.21 | 7.9 | 166.1 | 541.9 |  |  |  |  |  |
| UH36 | LL | 2460 ± 25 | LB shaft | F? | Adult | 16.3 | 8.1 | 46.6 | -17.9 | 3.3 | 0.21 | 10.6 | 173.2 | 578.8 |  |  |  |  |  |
| UH37 | PL | 2297 ± 25 | Ulna shaft | UK | Adult | 14.9 | 8.8 | 43.2 | -17.0 | 3.4 | 0.21 | 10.1 | 165.5 | 561.4 | 37 | 6.6 - 6.8 | 23734 | 0.70855 | 298.8 |

^a^ Later Lapita (LL), post-Lapita (PL), and late prehistoric/historic (LPH).

^b^ Analysed at the University of Waikato Radiocarbon Dating Laboratory (Hamilton, New Zealand).

^c^ Element sampled for stable isotope analysis. Unidentified long bone shaft fragment designated by ‘LB’.

^d^ Male (M), Female (F), adult of unknown sex (UK), and subadult (SA).

^e^ Years after birth (yrs), fetal weeks in utero (fwks), and child of unknown age (UKC).

^f^ Carbon and nitrogen stable isotope analysis was conducted by EA-IRMS at Iso-Analytical (Cheshire, UK) using a Europa elemental analyser and Europa 20-20 mass spectrometer. The internal standards IA-R005 (δ^13^C = -26.03 ‰) and IA-R006 (δ^13^C = -11.64 ‰) for carbon and IA-R045 (δ^15^N = -4.71 ‰) and IA-R046 (δ^15^N = 22.04 ‰) for nitrogen were analysed in sets of eight alongside the samples for quality control. Analytical precision was calculated from duplicate measurements of the samples and eighteen repeated measurements of the bovine liver control NIST-1577B (δ^13^C = -21.60 ‰ and δ^15^N = 7.65 ‰).

^g^ Bold and italicized samples did not reach the collagen quality criteria outlined in the text.

^h^ Sulfur stable isotope analysis was conducted by EA-IRMS (Europa elemental analyser and mass spectrometer) at Iso-Analytical (Cheshire, UK). Internal standards IAEA-SO-5 (δ^34^S = 0.50 ‰) and IA-R027 (δ^34^S = 16.30 ‰) were run in sets of six alongside the samples for quality control. Analytical precision was calculated from duplicate measurements of the samples and nine repeated measurements of the barium sulfate control IA-R036 (δ^34^S = 20.74 ‰).

^i^ Fédération Dentaire Internationale (FDI) two-digit system of designating teeth.

^j^ Estimated age of crown mineralisation.

^k^ Identification number for samples when analysed for ^87^Sr/^86^Sr at the Department of Human Evolution, Max Planck Institute for Evolutionary Anthropology (Leipzig, Germany).
