# Supplementary material for: Diet and Human Mobility from the Lapita to the Early Historic Period on Uripiv Island, Northeast Malakula, Vanuatu
Source: PLoS One. 2014 Aug 20;9(8):e104071. doi: 10.1371/journal.pone.0104071 (PMC4139273; doi:10.1371/journal.pone.0104071)
Supplement: Table S2 — Temporal period, bone collagen δ13C, δ15N, and δ34S values, and collagen quality indicators of the prehistoric pigs ( Sus scrofa ) from Uripiv. (DOCX) [file pone.0104071.s002.docx]

Table S2. Temporal period, bone collagen δ^13^C, δ^15^N, and δ^34^S values, and collagen quality indicators of the prehistoric pigs (*Sus scrofa*) from Uripiv.

| Lab ID | Period^a^ | %N^bc^ | δ^15^N (‰) | %C | δ^13^C (‰) | C:N | %S^d^ | δ^34^S (‰) | N:S | C:S |
| --- | --- | --- | --- | --- | --- | --- | --- | --- | --- | --- |
| UF1 | LPH | 14.06 | 7.68 | 41.60 | -20.06 | 3.5 |  |  |  |  |
| UF2 | PL | 14.26 | 7.68 | 41.71 | -19.25 | 3.4 |  |  |  |  |
| UF3 | PL | 13.17 | 7.16 | 39.09 | -17.87 | 3.5 | 0.21 | 12.42 | 140.81 | 487.63 |
| UF4 | PL | 16.47 | 5.94 | 47.18 | -19.14 | 3.3 | 0.19 | 8.76 | 196.67 | 657.27 |
| UF5 | LL | 13.03 | 7.72 | 38.49 | -17.27 | 3.4 |  |  |  |  |
| ***UF6*** | LL | ***10.41*** | ***6.69*** | ***42.00*** | ***-21.11*** | ***4.7*** |  |  |  |  |
| UF7 | LL | 14.95 | 7.24 | 43.10 | -18.29 | 3.4 | 0.18 | 11.79 | 186.55 | 627.48 |
| UF8 | LL | 14.40 | 7.80 | 42.24 | -17.28 | 3.4 |  |  |  |  |
| UF9 | Lapita | 13.32 | 8.64 | 40.16 | -16.91 | 3.5 |  |  |  |  |
| UF10 | Lapita | 15.26 | 6.72 | 45.16 | -19.43 | 3.5 |  |  |  |  |
| UF11 | Lapita | 15.12 | 7.24 | 43.88 | -18.54 | 3.4 | 0.22 | 12.83 | 160.24 | 542.49 |

^a^ Later Lapita (LL), post-Lapita (PL) and late prehistoric/historic (LPH ).

^b^ Carbon and nitrogen stable isotope analysis was conducted by EA-IRMS at Iso-Analytical (Cheshire, UK) using a Europa elemental analyser and Europa 20-20 mass spectrometer. The internal standards IA-R005 (δ^13^C = -26.03 ‰) and IA-R006 (δ^13^C = -11.64 ‰) for carbon and IA-R045 (δ^15^N = -4.71 ‰) and IA-R046 (δ^15^N = 22.04 ‰) for nitrogen were analysed in sets of eight alongside the samples for quality control. Analytical precision was calculated from duplicate measurements of the samples and eighteen repeated measurements of the bovine liver control NIST-1577B (δ^13^C = -21.60 ‰ and δ^15^N = 7.65 ‰).

^c^ Bold and italicized samples did not reach the collagen quality criteria outlined in the text.

^d^ Sulfur stable isotope analysis was conducted by EA-IRMS (Europa elemental analyser and mass spectrometer) at Iso-Analytical (Cheshire, UK). Internal standards IAEA-SO-5 (δ^34^S = 0.50 ‰) and IA-R027 (δ^34^S = 16.30 ‰) were run in sets of six alongside the samples for quality control. Analytical precision was calculated from duplicate measurements of the samples and nine repeated measurements of the barium sulfate control IA-R036 (δ^34^S = 20.74 ‰).
