# Supplementary material for: Diet and Human Mobility from the Lapita to the Early Historic Period on Uripiv Island, Northeast Malakula, Vanuatu
Source: PLoS One. 2014 Aug 20;9(8):e104071. doi: 10.1371/journal.pone.0104071 (PMC4139273; doi:10.1371/journal.pone.0104071)
Supplement: Table S3 — Plant type, sampling location, species, 87Sr/86Srplant ratios, and strontium concentration (Sr) of the modern plants analysed in this study. (DOCX) [file pone.0104071.s003.docx]

Table S3. Plant type, sampling location, species, ^87^Sr/^86^Sr_plant_ ratios, and strontium concentration (Sr) of the modern plants analysed in this study.

| Sample number^a^ | S-EVA ID^b^ | Sampling location^c^ | Island^d^ | Species | ^87^Sr/^86^Sr | Sr (ppm) |
| --- | --- | --- | --- | --- | --- | --- |
| SRP 4 | 25732 | Eratap | Efate | *Pandanus tectorius* | 0.7091 | 2611.5 |
| MRP 5 | 25733 | Eratap | Efate | *Persea americana* | 0.7090 | 764.5 |
| DRP 3 | 25731 | Eratap | Efate | *Psidium* sp*.* | 0.7090 | 449.4 |
| SRP 16 | 25736 | Emua | Efate | *Manihot esculenta* | 0.7065 | 459.6 |
| MRP 13 | 25734 | Emua | Efate | *Barringtonia* sp*.* | 0.7091 | 1701.8 |
| DRP 14 | 25735 | Emua | Efate | *Inocarpus fagifer* | 0.7064 | 855.0 |
| SRP 19 | 25738 | Epule | Efate | *Pandanus tectorius* | 0.7090 | 1760.5 |
| MRP 17 | 25737 | Epule | Efate | *Spondias dulcis* | 0.7088 | 349.3 |
| DRP 19 | 25739 | Epule | Efate | *Artocarpus altilis* | 0.7084 | 271.8 |
| SRP 26 | 25743 | Teouma inland | Efate | *Manihot esculenta* | 0.7073 | 430.2 |
| MRP 23 | 25740 | Teouma inland | Efate | *Psidium* sp*.* | 0.7083 | 336.5 |
| DRP 25 | 25741 | Teouma inland | Efate | *Artocarpus altilis* | 0.7069 | 136.1 |
| SRP 28 | 25745 | Teouma beach | Efate | *Colocasia esculenta* | 0.7087 | 160.1 |
| MRP 26 | 25742 | Teouma beach | Efate | *Hibiscus tiliaceus* | 0.7089 | 263.9 |
| DRP 28 | 25744 | Teouma beach | Efate | *Inocarpus fagifer* | 0.7086 | 108.1 |
| SRP 44 | 25748 | Uripiv | Uripiv | *Pandanus tectorius* | 0.7088 | 56.4 |
| MRP 42 | 25747 | Uripiv | Uripiv | *Barringtonia* sp*.* | 0.7088 | 2614.6 |
| DRP 41 | 25746 | Uripiv | Uripiv | *Mangifera* sp*.* | 0.7088 | 403.1 |
| SRP 45 | 25750 | Uripiv | Uripiv | *Musa* sp*.* | 0.7087 | 131.8 |
| MRP 45 | 25749 | Uripiv | Uripiv | *Citrus reticulata* | 0.7087 | 1375.7 |
| DRP 46 | 25751 | Uripiv | Uripiv | *Barringtonia* sp*.* | 0.7084 | 1827.2 |
| SRP 48 | 25754 | Kela's Garden | NE MK ML | *Cocos nucifera* | 0.7050 | 16.0 |
| MRP 47 | 25752 | Kela's Garden | NE MK ML | [*Thespesia populnea*](http://www.ctahr.hawaii.edu/forestry/trees/CommonTreesHI/CFT_Thespesia_populnea.pdf) | 0.7049 | 152.2 |
| DRP 47 | 25753 | Kela's Garden | NE MK ML | *Dracontomelon vitiense* | 0.7048 | 124.7 |
| SRP 53 | 25757 | Wala Mainland | NE MK ML | Bambuseae | 0.7102 | 7.7 |
| MRP 50 | 25755 | Wala Mainland | NE MK ML | *Hibiscus tiliaceus* | 0.7055 | 87.4 |
| DRP 50 | 25756 | Wala Mainland | NE MK ML | *Artocarpus altilis* | 0.7051 | 76.3 |
| SRP 57 | 25760 | Lek Lek | NE MK ML | *Musa* sp*.* | 0.7075 | 106.7 |
| MRP 54 | 25759 | Lek Lek | NE MK ML | *Theobroma cacao* | 0.7079 | 211.6 |
| DRP 54 | 25758 | Lek Lek | NE MK ML | *Dracontomelon vitiense* | 0.7076 | 72.6 |

^a^ Short-rooted plant (SRP), medium-rooted plant (MRP), and deep-rooted plant (DRP).

^b^ Identification number at the Department of Human Evolution, Max Planck Institute for Evolutionary Anthropology (Leipzig, Germany).

^c^ Refer to Figures 2 and 3.

^d^ Northeast Malakula Mainland (NE MK ML).
